# Effect of Quenching Tempering-Post Weld Heat Treatment on the Microstructure and Mechanical Properties of Laser-Arc Hybrid-Welded Boron Steel

**DOI:** 10.3390/ma12182862

**Published:** 2019-09-05

**Authors:** Ho Won Lee, Kwang Jae Yoo, Minh Tien Tran, In Yong Moon, Young-Seok Oh, Seong-Hoon Kang, Dong-Kyu Kim

**Affiliations:** 1 Department for Virtual Materials Processing, Korea Institute of Materials Science, Changwon 51508, Korea (H.W.L.) (K.J.Y.) (I.Y.M.) (Y.-S.O.) (S.-H.K.); 2 School of Mechanical Engineering, University of Ulsan, Ulsan 44610, Korea

**Keywords:** laser-arc hybrid welding, PWHT, boron steel, microstructure, mechanical properties

## Abstract

In the present study, we have investigated the effect of post-welding heat treatment (PWHT) of quenching and tempering (QT) on the microstructure and mechanical properties of welded boron steel joints processed using laser-arc hybrid welding on two commercial filler materials, SM80 (Type-I) and ZH120 (Type-II). The microstructure and mechanical properties of the weld joints were characterized via optical microscopy, Vickers microhardness, and the uniaxial tensile test. The macrostructure of the weld joint was composed of a fusion zone (FZ), heat-affected zone (HAZ), and base metal zone (BMZ). After the QT-PWHT, the QT specimens revealed the V-shape hardness distribution across the weld joint, while the as-welded specimen exhibited the M-shape hardness distribution. As a result, the QT specimens revealed the premature fracture with little reduction in the area at the interface between the HAZ and FZ, while the as-welded specimen exhibited the local necking and rupture in the BMZ. In addition, the Type-II filler material with a greater value of equivalent carbon content was rarely influenced by the tempering, maintaining its hardness in the as-quenched status, while the Type-I filler material showed a gradual decrease in hardness with the tempering time. The results demonstrate that the Type-II weld joint outperformed the Type-I weld joint in terms of the structural integrity of welded parts.

## 1. Introduction

Boron steel is one of the most widely used alloys for fabricating automotive components because of its good mechanical properties, welding performance, and hardenability [1]. It can be used for not only automotive chassis parts with a complex shape but also driving parts with severe abrasive conditions because of its good formability before the heat treatment and a high wear resistance and strength after quenching-tempering (QT) or press hardening. Boron is one of the main alloying elements used in boron steel to increase its hardenability. In addition, it ensures high strength, impact toughness, and good welding performance compared to carbon steel of similar strength [1].

Welding is a principal process for joining the structural metal products that are used in the automotive industry [2,3,4]. Advanced welding processes for automotive applications have been actively developed, enabling a reduction in vehicle weight. Recently, laser-arc hybrid welding has been treated as one of the most promising technologies based on the combination of advantages of arc welding and laser welding. Laser welding can achieve deeper penetration and higher welding speed due to the tight focus and high power of the laser beam. However, this method is very difficult for highly reflective materials with poor electrical efficiency and gap bridge ability, thus it requires high precision in the workpiece fit-up and edge preparation [5]. These drawbacks can be compensated for by using the arc welding process because of its advantages such as excellent gap bridging ability and high electrical efficiency regardless of reflectivity. Thus, laser-arc hybrid welding is a commonly applied technology in various manufacturing industries for the welding of metallic plates.

Many studies concerning hybrid welding technology have been conducted so far [6,7,8,9]. Zhang et al. [6] have succeeded in achieving a full penetration welded joint without visible flaws on the 40-mm thick plates of 316L austenitic stainless-steel by optimizing the laser-arc hybrid welding process. Kristiansen et al. [7] produced steady joints with adequate penetration for the flat position of the butt joint on the offshore steel by investigating the performance of different weldment types and welding positions in the hybrid laser-arc welding process. Son et al. [8] found that the hardness values of the heat-affected and fusion zones are higher than that of the base metal zone in laser-arc hybrid-welded boron steel. Gao et al. [9] reported that the hybrid welding had higher microhardness on the weld joint of a mild steel specimen compared to the arc welding.

The effect of a post-welding heat treatment (PWHT) on the microstructure and mechanical properties of weldments was studied intensively for enhancing its properties [10,11,12,13,14,15,16,17,18,19]. A PWHT is commonly used to reduce the residual stress formed during welding and improve the mechanical properties. Various techniques for heat treatment have been developed, such as annealing [10,11], tempering [12], quenching [13,14], and laser hardening [20,21]. Du et al. [10] applied a PWHT to the 2205DSS/Q235 joint to improve the toughness after laser-beam welding. The result showed that the high toughness of laser-beam-welded joints could be achieved at 500 °C and 600 °C due to the phase transformation from untempered to tempered martensite. Xin et al. [11] also investigated the effect of a PWHT on International Thermonuclear Experimental Reactor (ITER)-grade 316LN austenitic stainless-steel weldments by using a tungsten inert gas (TIG) welding technique. The combination of acceptable strength and high toughness was achieved on the laser-TIG hybrid weld joints [12]. Fadaeifard et al. [13] fabricated the weldment with remarkable enhancement in the yield strength and ultimate tensile strength associated with the indistinguishable microstructure of a TIG-welded AA6061-T6 alloy by applying a PWHT. Cho et al. [15] reported the effects of a PWHT on the residual stress in the multi-pass welds, which significantly decreased from 316 MPa to 39 MPa after a PWHT. Sadeghi et al. [16] presented a significant decrease of 35% in the residual stress in the as-welded specimen after a PWHT at 480 °C. Ma et al. [17] reported that the welded dissimilar metal joint exhibited the highest tensile strength of 890 MPa and elongation of 16% when the heat treatment temperature was at 400 °C. Guarino et al. [20] reported that improvement of the fatigue property can be achieved by applying the laser hardening technique, which is used for surface treatment with a high precision. The QT is frequently used for automotive parts made of boron steel and it is also highly required for welded boron steel parts such as a light-weight hollow drive shaft [22]. To date, however, there have been few studies on the effect of QT-PWHT on the microstructure and mechanical properties of laser-arc hybrid weld joints.

In this study, we investigated the effect of the QT-PWHT on the microstructure and mechanical properties of welded boron steel joints processed using laser-arc hybrid welding on two commercial filler materials, SM80 (Type-I) and ZH120 (Type-II). The QT was applied to the butt-welded joints made from boron steel plates with a thickness of 4.5 mm. The microstructure and mechanical properties of the weld joints were characterized using optical microscopy, Vickers microhardness, and a uniaxial tensile test. We discuss the correlation of the local phase transformation due to the QT-PWHT with the variation in the location of the tensile fracture and hardness distribution in a comparison between Type-I and Type-II weld joints.

## 2. Experimental Procedure

### 2.1. Materials

In this study, HS35B20 boron steel plate with a thickness of 4.5 mm was prepared as a base metal for the laser-arc hybrid welding process. The welding process was conducted using two commercial filler materials, SM80 (Type-I, Hyundai welding, Ulsan, korea) and ZH120 (Type-II, KISWEL, Seoul, Korea). The reason for choosing these filler materials was to compare the effects of QT-PWHT under different welding materials. Table 1 presents the chemical compositions of the base metal and filler materials. The equivalent carbon contents calculated using the equation by Ito and Bessyo [23] were 0.19 wt% (Type-I), 0.25 wt% (Type-II), and 0.44 wt% (base metal).

### 2.2. Laser-Arc Hybrid Welding and QT-PWHT

**Figure 1:**
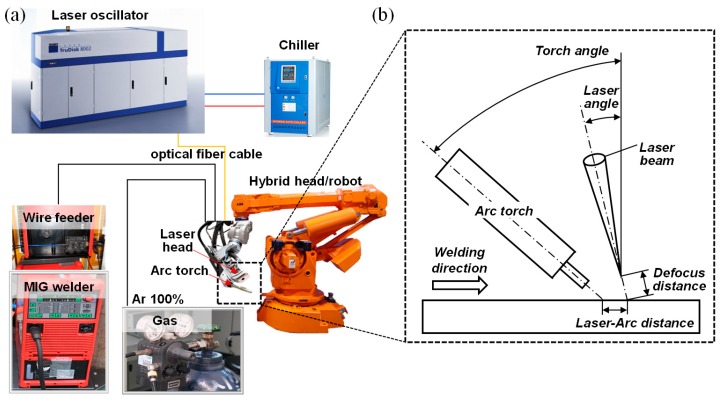
(**a**) Laser-arc hybrid welding apparatus and (**b**) schematic of the process.

The boron steel plates were butt-welded using a laser-arc hybrid welding process. The experimental set-up was composed of a TruDISK 8002 disk laser (TRUMPF, Ditzingen, Germany) and a TPS5000 arc power source (Fronius, Pettenbach, Austria) equipped with an IRB6400 robot (ABB, Zurich, Switzerland) (Figure 1). The welding experiments were carried out on a hybrid welding system classified as laser-metal inert gas (MIG) welding. In this investigation, the laser preceding condition was applied to introduce the weld joints. The system was equipped with 100% Ar at a flowing rate of 21 L/min as shielding gas. The objective of the Ar gas was to protect the molten fusion zone from atmospheric contamination. The laser beam was produced by using a laser power of 3900 W as the main heat source during the laser-arc hybrid welding to maintain the deep penetration. An optical fiber with a diameter of 600 μm was used in the welding process. The incidence angle of the laser beam with the irradiated specimen surface was fixed at 5° along the welding direction to avoid any damage of the laser head because of the reflected beam. The wire feed rate was selected to be 5 m/min with the current and the voltage set at 198 A and 18.5 V, respectively, in the arc welding system. The distance between the laser and arc was 4 mm, which affected the morphological characteristics of the fusion zone [5]. The defocus distance of the laser beams and the stick-out from the edge of the specimen to welding torch were 10 mm and 14 mm, respectively. Table 2 presents the processing parameters used in the laser-arc hybrid welding. After the hybrid welding process, the welded joints were then subjected to the QT. They were heated at 870 °C for 1 h, followed by water quenching to room temperature and then tempered at 180 °C for 1 or 2 h followed by air cooling. The specimens were then polished using a SiC paper, diamond suspensions, and followed by colloidal silica. After the mechanical polishing, the specimens were etched using a 3% HNO_3_ + C_2_H_5_OH solution in order to reveal their microstructure.

### 2.3. Mechanical Testing

**Figure 2:**
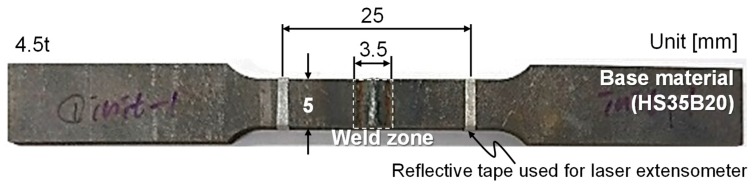
Tensile specimen (ASTM E8M-11).

**Figure 3:**
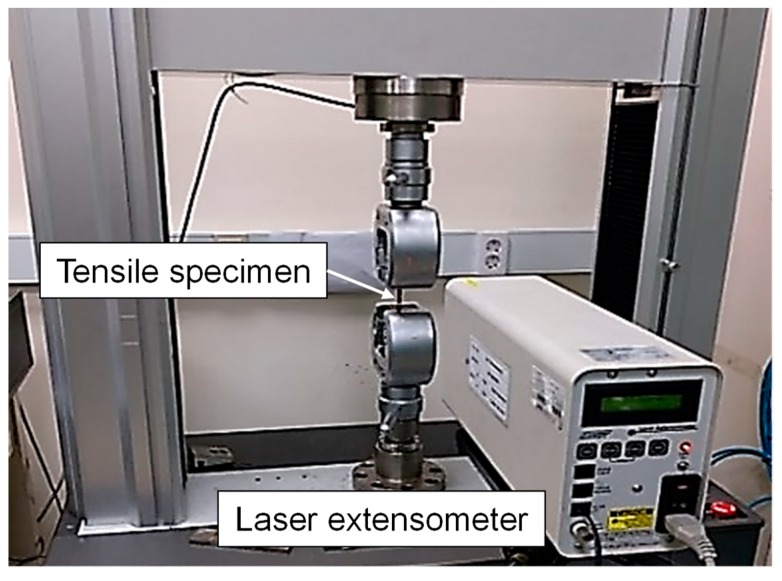
Experimental setup of the uniaxial tensile testing.

**Figure 4:**
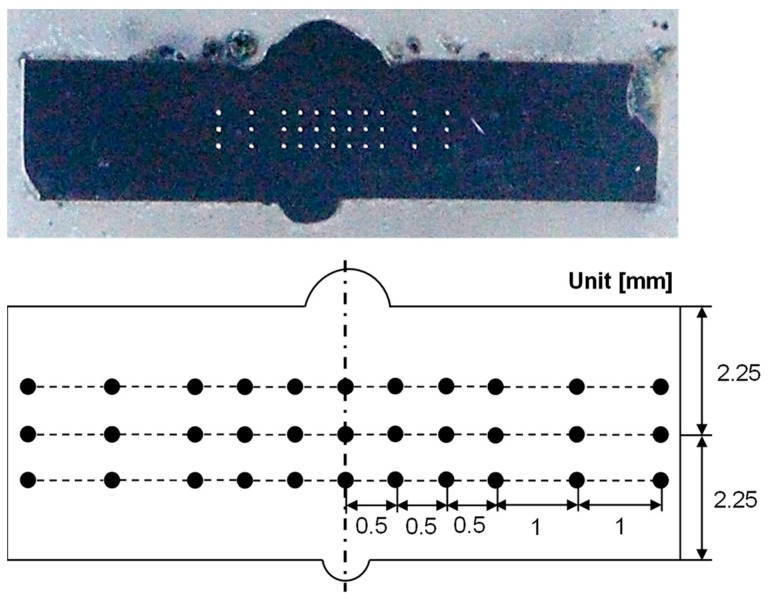
Vickers microhardness measurement points.

The uniaxial tensile tests were carried out to evaluate the mechanical properties of butt-welded boron steel before and after the QT process. The specimens were fabricated in accordance with American Society for Testing and Materials (ASTM) E8M-11, as shown in Figure 2. The gauge length and the width of the specimen used were 25 mm and 5 mm, respectively. Figure 3 shows the experimental setup for the tensile test using a laser extensometer at room temperature with a ram velocity of 0.001 mm/s. The tensile tests were repeated three times for each condition. Figure 4 shows the Vickers microhardness measurement points. The indentation load was 1 kgf with a dwell period of 10 s.

## 3. Results and Discussion

**Figure 5:**
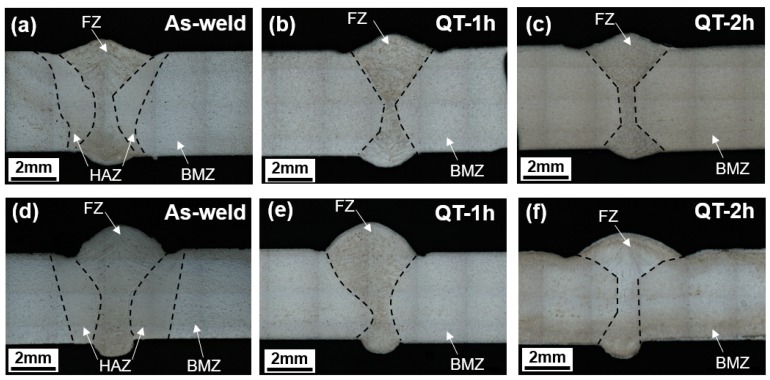
Macrostructures of the laser-arc hybrid weld joints: (**a**–**c**) Type-I and (**d**–**f**) Type-II, (FZ: fusion zone, HAZ: heat affected zone, BMZ: base material zone).

Figure 5 shows the macrostructures of the laser-arc hybrid weld joints. The macrostructure of the weld joint was composed of the three distinct regions of the base material zone (BMZ), heat-affected zone (HAZ), and fusion zone (FZ). As shown in the figure, the HAZ in both Type-I and Type-II weld joints disappeared after QT-PWHT.

**Figure 6:**
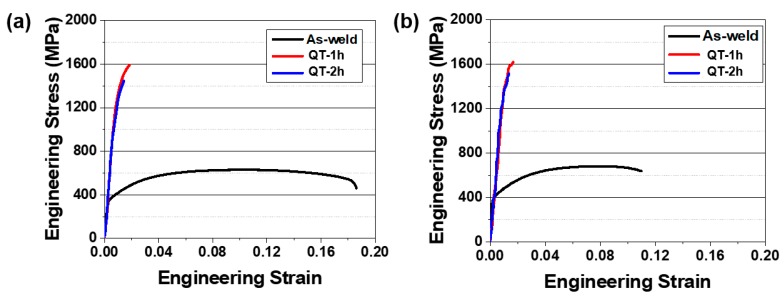
Engineering stress–strain curves determined from the uniaxial tensile test for the laser-arc hybrid weld joints: (**a**) Type-I and (**b**) Type-II.

Figure 6 shows the engineering stress–strain curves measured from the uniaxial tensile test for the Type-I and Type-II weld joints. After the QT-PWHT, it revealed substantial changes in the stress–strain curve; an increase in the flow stress and a decrease in the elongation to fracture. In comparison between the Type-I and Type-II weld joints, the as-welded specimens showed an apparent difference especially in the elongation to fracture. Specifically, the Type-I welds showed an initiation of yielding at 360 MPa (*σ_y_*, yield strength), followed by significant uniform elongation to a peak stress of 630 MPa ($\sigma_{UTS}$, ultimate tensile strength) and a noticeably long post elongation. The Type-II weld showed a higher level of yield stress at 400 MPa (*σ_y_*) and early diffuse necking at 682 MPa ($\sigma_{UTS}$) with a relatively small amount of plastic deformation. After the QT-PWHT, both types of weld joints exhibited quite similar stress–strain behaviors with significantly high strength and little plastic deformation, indicating the typical brittle fracture.

**Figure 7:**
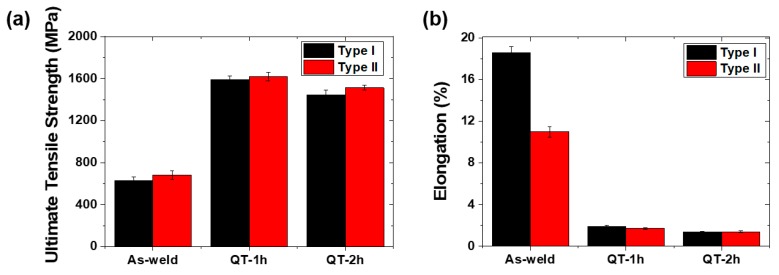
(**a**) Ultimate tensile strength and (**b**) elongation to fracture determined from the uniaxial tensile test for the laser-arc hybrid weld joints.

Figure 7 summarizes the ultimate tensile strength and elongation to fracture of the laser-arc hybrid weld joints. After the QT-PWHT, both types of weld joints showed an increase in the ultimate tensile strength (UTS) of ≈135%, while the Type-I and Type-II weld joints exhibited a decrease in the elongation to fracture of −91% and −86%, respectively. Specifically, as shown in Figure 7a, the UTS of 630 MPa for the Type-I weld increased up to 1592 MPa for the tempering time of 1 h and then slightly decreased to 1446 MPa for the tempering time of 2 h. Furthermore, the UTS of 682 MPa for the Type-II weld increased enormously up to 1619 MPa for the tempering time of 1 h and then decreased to 1513 MPa for the tempering time of 2 h. The elongation to fracture, however, revealed a monotonic decrease with the tempering time, as shown in Figure 7b. The elongation to fracture of 18.6% for the Type-I weld decreased to 1.9% and 1.4% for the tempering times of 1 and 2 h, respectively. Furthermore, the elongation to fracture of 11% for the Type-II weld decreased to 1.7% and 1.4% for the tempering times of 1 and 2 h, respectively.

**Figure 8:**
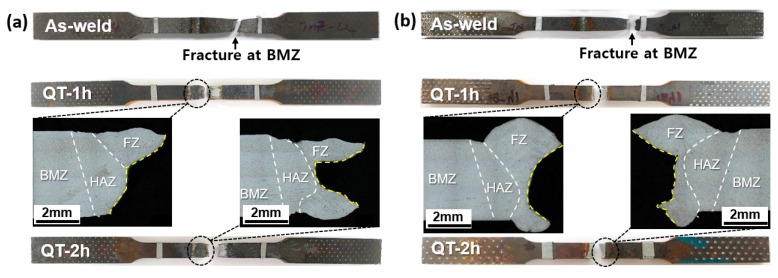
Fractured tensile specimens for the laser-arc hybrid weld joints: (**a**) Type-I and (**b**) Type-II.

Figure 8 shows the fractured tensile specimens for the laser-arc hybrid weld joints. The fracture occurred in the BMZ for the as-welded specimens, while it took place at the interface between the HAZ and FZ after the QT-PWHT. In addition, the as-welded specimens showed a noticeable diffuse necking due to plastic localization, while the QT specimens exhibited a premature fracture with little reduction in the area. It is conceivable that the local variation of mechanical properties was responsible for the position and mode of tensile fracture. Specifically, the premature brittle fracture could be attributed to the stress concentration arising from the steep gradient of strength and hardness across the interface [24,25,26]. 

**Figure 9:**
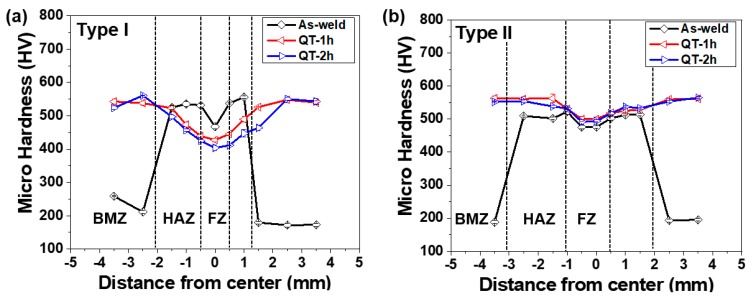
Microhardness distribution along with the mid-thickness layer of the weld joints: (**a**) Type-I and (**b**) Type-II.

Figure 9 shows the hardness distribution along with the mid-thickness layer of weld joints before and after the QT-PWHT process. The distribution of hardness was apparently inhomogeneous from the BMZ to the FZ. The as-welded specimen revealed the M-shape distribution across the weld joint, while the QT specimens exhibited the V-shape distribution due to the significant increase of ≈170% in the BMZ after the QT-PWHT. Before the QT-PWHT, there was no significant difference in the distribution of hardness between the Type-I and Type-II weld joints. The hardness in the BMZ for both weld joints was ≈190 HV. The average hardness value of ≈550 HV in the HAZ and the value for the FZ was twice as hard as the one in the BMZ. Therefore, the local necking and rupture happened in the BMZ for both as-welded joints, as shown in Figure 8.

The hardness distribution changed drastically after the QT-PWHT. In Figure 9a, the hardness for Type-I in the BMZ and HAZ increased significantly compared to that of the as-welded joint while the hardness in the FZ decreased gradually with the tempering time compared to the as-welded specimens. In Figure 9b, the change of hardness distribution for Type-II showed a different tendency compared to that of Type-I. After the QT-PWHT, the hardness for Type-II increased over all regions and its distribution became relatively uniform, resulting in the average hardness of ≈540 HV, regardless of the tempering time. The reduction in hardness with the tempering time was negligible and the hardness remained higher than that of the as-welded specimen. Thus, the hardness for the Type-II weld joint was less affected by the QT-PWHT compared to that of the Type-I joint. Therefore, it can be concluded that the Type-II filler material is more preferable for a welded structure that requires high hardness and local strength for the application to automotive drive parts [24].

**Figure 10:**
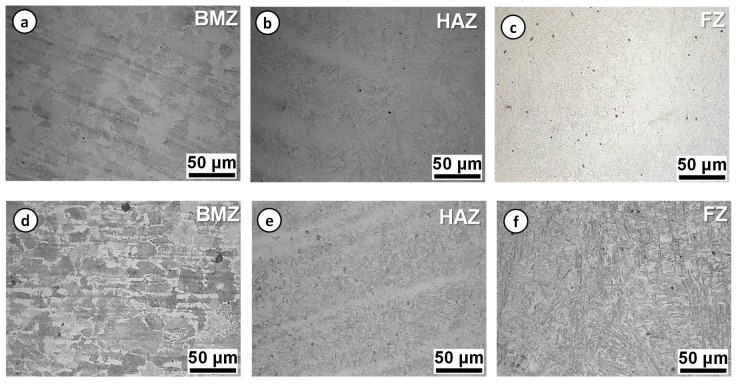
Microstructures of laser-arc hybrid weld joints: (**a**–**c**) Type-I and (**d**–**f**) Type-II.

Figure 10 shows the microstructures of the as-welded specimens fabricated using the Type-I and Type-II filler materials. The microstructure in the BMZ was composed of the ferrite-pearlite structure, which is a typical phase of the rolled boron steel. The microstructures in the HAZ and FZ were mainly the martensite phase, which was transformed from the austenite phase during the welding process. The constituent phases in the FZ were expected to have little ferrite and martensite for Type-I and fully martensite for Type-II based on Schaeffler’s diagram [27]. Indeed, it was revealed that the constituent phases in the HAZ and FZ of the as-welded specimen were mainly comprised of martensite and bainite in the previous study [8].

The equivalent contents of chromium and nickel were 1.51 wt% and 2.61 wt% for Type-I and 1.35 wt% and 5.96 wt% for Type-II: Ni_eq_ = %Ni + 30 × %C + 0.5 × %Mn and Cr_eq_ = %Cr + %Mo + 1.5 × %Si + 0.5 × %Nb. Such a local variation in the microstructure resulted in the M-shape hardness distribution across the weld joint for the as-welded joints, as seen in Figure 9. 

**Figure 11:**
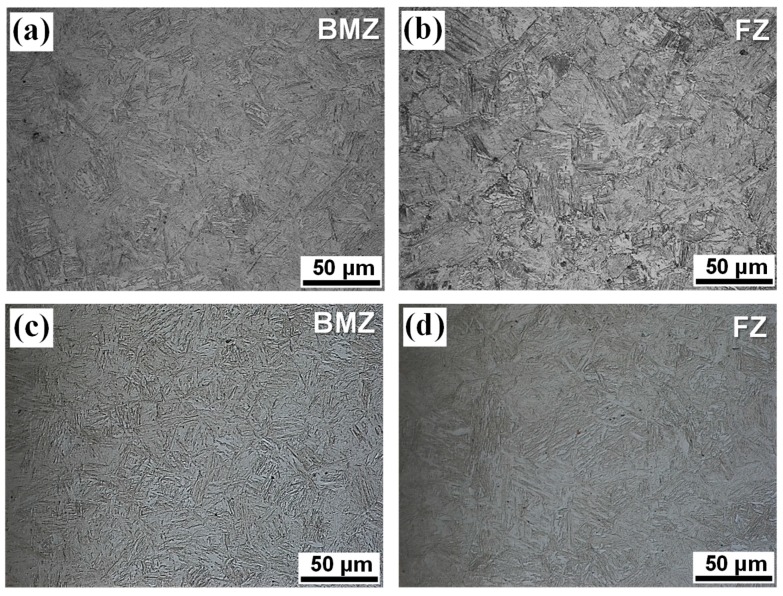
Microstructures of laser-arc hybrid weld joints after the tempering time of 2 h: (**a**,**b**) Type-I and (**c**,**d**) Type-II.

Figure 11 shows the microstructures after the QT-PWHT for the tempering time of 2 h. In the QT-PWHT, when heated at 870 °C for 1 h, the ferrite-to-austenite phase transformation took place in the BMZ and the austenite phase changed into the martensite phase when quenched in water due to a rapid cooling rate. The microstructural change from a ferrite-pearlite structure to the martensite resulted in a drastic increase in hardness in the BMZ. When tempered at a low temperature of 180 °C, tempered martensite appeared in the BMZ and FZ for both types of weld joints. It is noted that the Type-II filler material had a greater value of equivalent carbon content than that of the Type-I filler material. The equivalent carbon content calculated using the equation from Ito and Bessyo [23] was 0.19 wt% and 0.25 wt% for the Type-I and Type-II filler materials, respectively. The boron steel in the BMZ had a greater amount of carbon content of 0.44 wt% compared to the filler materials. Since the weld with a higher carbon content generally has the greater hardness [28], it resulted in the V-shape hardness distribution across the weld joint after the QT-PWHT, as seen in Figure 9. In addition, the Type-II filler material was hardly influenced by the tempering compared to the Type-I filler material, maintaining the hardness of the as-quenched state.

## 4. Conclusions

In the present study, we have investigated the effect of a post-welding heat treatment (PWHT) of quenching and tempering (QT) on the microstructure and mechanical properties of welded boron steel joints processed using laser-arc hybrid welding on two commercial filler materials.

(i) For the as-welded specimen, the microstructure in the BMZ was composed of the ferrite-pearlite structure, while the microstructures in the HAZ and FZ were mainly in the martensite phase. Such a local variation in the microstructure resulted in the M-shape hardness distribution across the weld joint, leading to the local necking and rupture in the BMZ under the uniaxial tension. 

(ii) For the QT specimen, the tempered martensite appeared in the BMZ and FZ for both types of weld joints. The martensite phase in the BMZ, with a greater amount of carbon content compared to the HAZ and FZ, resulted in the V-shape hardness distribution across the weld joint, giving rise to premature fracturing with little reduction in the area at the interface between the HAZ and FZ under the uniaxial tension. 

(iii) In addition, the Type-II filler material with a greater value of equivalent carbon content was rarely influenced by the tempering, maintaining the hardness of the as-quenched state, while the Type-I filler material showed a gradual decrease in hardness with the tempering time. The results demonstrate that the Type-II weld joint outperformed the Type-I weld joint in terms of the local variation of hardness.

## Figures and Tables

**Table 1:** Chemical composition of materials (wt%).

Materials	C	Mn	Si	Cr	Ti	B	P	S	Ni	Mo	Fe
**Base metal (HS35B20)**	0.35	1.3	0.2	0.2	0.02	0.002	0.015	0.002	-	-	Bal.
**Type-I filler (SM80)**	0.056	1.85	0.81	0.025	0.15	-	0.018	0.007	0.005	0.27	Bal.
**Type-II filler (ZH120)**	0.06	1.48	0.52	-	-	-	0.002	0.003	3.42	0.57	Bal.

**Table 2:** Processing parameters used in the laser-arc hybrid welding.

Flowing Rate (L/min)	Laser Power (W)	Laser Speed (m/min)	Wire Feed Rate (m/min)	Current (A)	Voltage (V)	Laser-Arc Distance (mm)	Defocus Distance (mm)	Laser Angle (°)	Torch Angle (°)
21	3900	1.8	5	198	18.5	4	10	5	45
